# The Cholera

**Published:** 1849-02

**Authors:** 


*The Cholera.*—With every reasonable prospect of a third visitation of this fearful disease during the ensuing year, the question of its communicability becomes one of considerable importance. If the disease be contagious, it is important to know under what circumstances this property may be mitigated, or possibly destroyed, or if solely dependant for its propagation on atmospheric conditions, how far we possess the means of neutralizing them. These questions are of exceeding moment, both here and elsewhere. The experience of the past must be our guide for the future. Erratic, although the disease has manifested itself in its course, and capricious, if we may use the term, in the selection of its places of visitation, yet careful observation has disclosed many circumstances which favour its development, and knowing these, we may have it in our power to diminish its malignity and to restrict the number of its attacks.

The disease is by no means one of recent origin. The description of a disease of an analogous character, if not identically the same, has been described in a Hindoo work of great antiquity; and between the years 1629 and 1781, repeated epidemics of a disease approaching in its character to Algide or Asiatic cholera, have been described as having visited India and Hindostan. In the latter year the disease fatally visited Ganjam, a city of

Hindostan, situated on the Bay of Benegal, and destroyed in this and other cities, in a short period of time 30,000 negroes, and 8,000 of the white population.—Whatever may have been the origin of the disease in those days, its ravages were of a local character, and although it must have prevailed epidemically, yet we have no account of its having traveled beyond the countries specified. This may very possibly have been due to the more restricted international intercourse which then existed. One thing is certain, that with the solitary exception of an epidemic of this disease which prevailed throughout Europe towards the close of the seventeenth century, it has restricted its ravages to the countries specified, prevailing in them at different times and different places, with marks occasionally of a sporadic, at other times of an epidemic character, and continued to do so till the memorable year 1817, when it manifested itself in Jessore, a city of British India, situated on the Delta of the Ganges, whence it spread, like a destroying angel, to the south, north, east, and the west, proving equally fatal and malignant everywhere, and unchecked in its career, either by the severity of winter, or the expanse of the Atlantic ocean, only ceased its ravages on the confines of civilization in this Hemisphere. During this period of fifteen years, its march appeared to be a steady and an onward one. In 1819, it penetrated to its most southerly point, invading the Mauritius, in 20° south latitude. In 1829, it reached Archangel, on the White Sea, in 64° north latitude: the most easterly direction of which we have account was the Philippine Islands, situated in east longitude 125°, which it invaded in 1831; and its most westerly, St. Louis, Missouri, in 1832, situated in about 90° of west longitude; thus running over, during the years specified, no less than 84° of latitude, and 215° of longitude. Such was the disease which originated at Jessore in 1817. In 1845–'6 it again broke out at Curachee, a town situated near the mouth of the Indus. During the past and the present year, it has visited the principal kingdoms of Europe, with a rapidity seven fold more quick, and the history of its westward progress, is an object of intense anxiety.

A careful examination of all the evidence with reference to the origin and progress of the cholera, discloses this important fact, that a humid atmosphere, wet and sultry weather, and marshy situations, are peculiarly adapted to its development. Exceptions will undoubtedly be found to the complete truthfulness of this observation, but in its main features the observation will hold good, and may be safely acknowledged as a rule. In 1817, the summer was a peculiarly rainy one at Jessore, and the city itself is surrounded by marshes. In 1846, Dr. Thom, of the 86th Regiment, stationed at Curachee, observes that "the thermometer stood at from 98° to 104° Fahrenheit, and the quantity of moisture was greater than I ever saw in any part of the world, at any season, the dew point being at 83°, and the thermometer in the shade being at 90°, the lowest range; even this gives 12·19 grains of vapor in each cubic foot of air," and he further shows that the quantity of rain which fell was unusually great. When the epidemic raged in Burmah, Dr. Parke observes—"during its progress, it attacked chiefly or exclusively the towns and villages situated in low and marshy places, on the banks of rivers and shores of the sea." In India and Hindostan, it was observed to prevail most frequently with southerly or easterly winds, which favored moisture, and as a general rule, we may observe, that this excessive moisture was either a prelude to, or an accessory of, its appearance, as witnessed by Dr. Prout, during its existence in England in 1831–'2; and, wherever it has prevailed, this fact is notorious, that the most marshy situations, the worst drained localities, have been especially selected as the sites of its greatest virulence. Whether all this induces a cause of malarial origin, of electrical atmospheric disturbances, or whether this state of the atmosphere predisposes to the generation of animalculæ or fungoid causes of the disease, is a matter of little moment, as regards the lesson obviously taught. Although exceptions are to be found of its prevalence in dry and arid situations, yet they are too few to invalidate the above position as the rule.

Of what nature soever be the exciting cause of this disease, and there has been no want of speculation on this point, its mode of propagation is a question of at least as great, if not greater, importance. Does the disease propagate itself by contagion, or is it a simple epidemic of a non-contagious character? The medical world has been much divided on these two questions. When we reflect that contagious diseases frequently exhibit themselves in a form apparently epidemic, and that epidemics assume many of the features of contagious diseases, it becomes a matter of exceeding difficulty to draw the line of demarcation between them. We do not mean to assert that epidemics are necessarily contagious, or that contagious diseases are necessarily epidemic, but we mean to say that with reference to cholera, generalizations have been formed and conclusions arrived at without a full and attentive consideration of all the facts of the case. A reversion of opinion has taken place in favor of its contagious character, even among the most strenuous non-contagionists. It is not our intention to enter upon, or discuss the data upon which these conclusions have been arrived at; that would form matter for a whole number of our journal. But we may contrast, not without some degree of interest, the altered opinions of one of the most authoritative boards on the subject in Great Britain. At the last visitation of cholora in England, the Metropolitan Sanitary Commission emphatically declared the disease to be non-contagious; this year the General Board of Health, of London, treats this question in the most cautious non-committal way, observing that "the extent," uniform tenor, and undoubted authority of the evidence obtained from observers of all classes in different countries, etc., *appears to discredit* the once prevalent opinion, that cholera is *in itself* contagious, an opinion which, if fallacious, must be mischievous. And again, "It is so far true that certain conditions may favor its spread from person to person," etc., etc., etc. The Central Board of Health, again, of Dublin, is equally cautious. While, in one portion of its address, it talks of the "*non-contagious character of cholera*," in another it says, equally distinctly, "*that it is rarely, if ever, contagious*"—a species of phraseology which, to our mind, is sufficiently conclusive as to its being so sometimes. While evidence of the strongest description is adduceable to prove its epidemic character, evidence equally conclusive can be produced to demonstrate that it is contagious—contagious, however, under like circumstances with typhus fever or dysentery, but not to the same extent; and the means capable of depriving the latter of much of their malignity in this respect, are equally, if not more effectual with the former. We have a decided objection to conceal the truth in this matter, fully persuaded that ignorance of causes does infinitely more harm than their divulgence. It lulls into a security which is false, and prevents a recourse to precautionary measures, which would otherwise, in all probility, have obviated an attack. This city has much less to fear from a third visitation than what it had on the first or second. For cleanliness, it is now, probably, without a parallel on this continent. Still, there is a good deal yet to be done in the way of drainage, one of its most effectual preventives; for it has been well observed, "that in a locally impure atmosphere, individuals are attacked in a greater proportion than other members of the community."—*British Am. Jour. of Med. and Phys. Sci.*

